# Group A Streptococcus infections in children and adolescents in the post-COVID-19 era: a regional Italian survey

**DOI:** 10.1186/s13052-024-01750-6

**Published:** 2024-09-16

**Authors:** Bianca Laura Cinicola, Ilaria Sani, Federica Pulvirenti, Martina Capponi, Fabrizio Leone, Alberto Spalice, Agata Montalbano, Alessandra Macari, Maria Teresa Fonte, Paolo Gianni Giampietro, Danilo Buonsenso, Anna Maria Zicari, Teresa Rongai, On behalf of the Italian Pediatrician Federation (FIMP), Lazio Region, B Baldini Ferroli, R Brugnoli, FM Carpita, G Caruso, C Castellano, C Cives, L Costabile, R D’agostino, V De Vittori, A Fostira, C Grassi, I La Bella, AM Le Pera, A Masetti, D Morano, C Pontesilli, A Ragno, L Reali, PL Rotili, J Serafinelli, S Triarico, E Zirletta

**Affiliations:** 1 Department of Maternal Infantile and Urological Sciences, Sapienza University of Rome, Rome, Italy; 2 Department of Molecular Medicine, Sapienza University of Rome, Rome, Italy; 3 Primary Care Pediatrician, FIMP Roma, Rome, Italy; 4 Reference Centre for Primary Immune Deficiencies, AOU Policlinico Umberto I, Rome, Italy; 5 Primary Care Pediatrician, FIMP Viterbo, Viterbo, Italy; 6 Department of Woman and Child Health, Fondazione Policlinico Universitario A. Gemelli IRCCS, Rome, Italy; 7 Center for Global Health Research Studies, Università Cattolica del Sacro Cuore, Rome, Italy

**Keywords:** Group A Streptococcus infection, GAS, Children, Pharyngitis, Antibiotics, Influenza, COVID-19, Pandemic

## Abstract

**Background:**

Despite the worldwide increasing incidence of Group A Streptococcus (GAS) infections reported since December 2022, data on noninvasive GAS (nGAS) infections in the post COVID-19 era are limited. By a self-reported survey performed in an outpatient setting, we investigated the number and clinical features of GAS infections, the diagnostic work-up and the type of treatment utilized. In addition, the rate of influenza vaccination was evaluated.

**Methods:**

In June 2023 family pediatricians involved in the study sent the survey to parents of patients aged 0–16 years. The survey included questions on GAS infections that occurred from January 1 to May 31, 2023.

**Results:**

Among 3580 children, 20.3% had a GAS infection (0,8% < 1 year, 16,4% 1–3 years, 42,3% 3–6 years, 26,5% 6–9 years, 11,4%, 9–12 years, and 2,6% 12–16 years). Symptoms reported were sore throat (76.9%), fever (75.2%), tonsillar exudate (25.2%), lymphadenopathy (21.8%), and scarlet fever (14.7%). A single patient was hospitalized due to GAS meningitis.

Twenty four percent of children had more than one GAS infection. In this group, frequencies of symptoms reported in the first and in the following infection were similar, except for fever and scarlet fever which were less frequent during relapses. GAS was identified by rapid antigen detection test in 81.0% of children. Eighty-nine per cent of children were treated with antibiotics, mostly amoxicillin/clavulanate (40.4%) and amoxicillin (39.4%). Thirty four percent of children received influenza vaccine. No difference was observed among immunized and not immunized regarding the number and characteristics of GAS infection.

**Conclusions:**

We reported a certain prevalence of nGAS infections in children, mainly those aged 3–6 years age, who were mostly characterized by a low score of symptoms, and in most of the cases diagnosed and treated using a microbiological test as confirmatory tool.

In this new clinical setting, a national study would be useful to reach more significant data for the definition of a correct diagnosis and clinical management of nGAS infections in children. Moreover, it is important to improve flu vaccination campaign and coverage to protect children from coinfections that could worsen the disease and misdiagnose the etiology of pharyngitis.

**Supplementary Information:**

The online version contains supplementary material available at 10.1186/s13052-024-01750-6.

## Background

*Streptococcus pyogenes*, also called Lancefield group A streptococcus (GAS), is a ubiquitous gram-positive coccus that can colonize the human skin and the nasopharynx. However, multiple subtypes exist and differ in terms of the virulence factors responsible for the GAS pathogenesis and the broad clinical spectrum of disease [1].

Epidemiologically, GAS can be classified into more than 220 emm (M) types, based on the amino terminal gene sequence of the surface-exposed M protein, which shows different patterns of regional and global distribution [2].

Streptococci typically account for approximately one-third of acute pharyngitis cases, with the majority being secondary to viral pathogens [3]. The pharynx of children represents the primary GAS reservoir, spreading in temperate climates mostly in winter and spring. Transmission generally occurs via droplets, leading mainly to superficial or non-invasive infections (nGAS), such as pharyngitis and impetigo, or locally invasive skin infections and local suppurative complications. GAS is unfrequently responsible for invasive infections (iGAS) and for toxin-mediated or immune-mediated manifestations [4], causing almost 500 000 deaths worldwide every year [5]. Despite being mainly benign, if not promptly and adequately treated, GAS infection can develop severe disease and sequelae, as in the case of acute rheumatic fever, which severely impacts health and economic burden [6, 7].

During the COVID-19 pandemic, containment measures for SARS-CoV-2 diffusion, led to a reduction in the incidence of respiratory infections [8], including GAS disease [9]. However, since December 2022, an unusual increase in incidence rates of scarlet fever and iGAS infections has been reported by several countries across Europe, together with a relevant number of deaths in children under 10 years of age [10–15]. It has been hypothesized that the observed increase could be attributed to a lack of immunity due to reduced GAS exposure especially in younger children. The concomitant increase in respiratory syncytial virus (RSV) and seasonal influenza might also have contributed to the development of complicated forms [13, 16].

Despite the generally increasing incidence of GAS infections, data on nGAS infections in the post COVID-19 era are limited. Indeed, epidemiological studies capturing the full spectrum of GAS disease are challenging. In Italy, GAS surveillance only involves notification in cases of scarlet fever and invasive forms, and it is not possible to track the overall number of nGAS infections.

Here, we used a self-report survey to investigate the number and clinical features of nGAS observed in the first five months of 2023. The survey was promoted by family pediatricians working in the Italian territory, who diagnose and manage uncomplicated infectious diseases daily and were among the first to observe the increasing diffusion of GAS infections affecting young patients.

In this study, we aimed to assess the prevalence of GAS infections and to characterize the clinical manifestations, relapse of infections, diagnostic work-up and treatment of children belonging to the Lazio Region, in the middle of Italy. In addition, since influenza coinfection could be a risk factor for the development of a severe GAS disease or complications, the rate of influenza vaccination was also investigated.

## Methods

### Study population and design

Italian family pediatricians of FIMP (Federazione Italiana Medici Pediatri- Italian Pediatrician Federation), working in the Lazio Region, were involved in the study. In May 2023, those pediatricians were invited to participate via email. Clinicians who agreed to participate, sent the survey to all the parents/caregivers of the children they assisted (aged 0-16 years) through the messaging service Pediatotem^®^-Lviiier srl, a digital platform developed to conduct epidemiological studies among family pediatricians and parents (https://pediatotem.it/home/). The survey included questions on GAS infections that occurred from January 1 to May 31, 2023. If a recurrent GAS infection, but not the first infection, occurred during the study period, the participant was excluded from the analysis. The first and second reminders were sent by email 14 days and one month later, respectively. Replies were allowed until 15th July 2023. Written informed consent from the participants’ legal guardians/next of kin was obtained in accordance with the national legislation and institutional requirements.

### Survey development and content

The survey is available in Supplementary, Appendix Table A1 (Italian version) and Supplementary, Appendix Table A2 (English translation for editorial purposes).

### Statistical analysis

All the data were extracted on July 30, 2023. Continuous variables have been described using median and interquartile ranges (IQRs), and categorical variables using frequencies and percentages. Comparisons of continuous parameters between treatment groups were performed with a t-test if normally distributed and with a Mann-Whitney U test if not normally distributed (as tested by the Kruskal-Wallis test); differences in frequencies between groups were calculated by using the χ2 exact test. Differences were considered significant when p values were < 0.05. Statistical analyses were performed with SPSS 25.0 software for Windows (SPSS, Chicago, IL, USA).

## Results

From June 1 to July 15, 2023, the survey was sent to 196 possible family pediatrician respondents. Twenty-eight agreed to participate (27 from the city of Rome and one from Viterbo). Data on 3580 children (F/M ratio 0.86, median age of 5.4 years (IQR 3.3–7.5 years), were collected. Three hundred twenty-five patients were aged less than one year (9.1%), 723 were aged 1–3 years (20.2%), 924 3–6 years (25.8%), 730 6–9 years (20.4%), 547 9–12 years (15.3%), and 331 were aged 12–16 years (9.2%). The characteristics of participants are summarized in Table 1.

Seven hundred twenty-five children (20.3%) were reported to have been infected by GAS. Specifically, 0,8% of the children with GAS were aged less than one year, 16,4% 1–3 years, 42,3% 3–6 years, 26,5% 6–9 years, 11,4%, 9–12 years, and 2,6% 12–16 years. Eighty one parents answered our questions regarding one or more children in the same family, for a total of 170 cases.

GAS was identified by rapid antigen detection tests in 81.0% of the children, by throat culture swabs in 7.8%, and by clinical signs in 11.2%.

**Table 1:** Characteristics of participants

	Study participants *N* = 3580	
Characteristics	*n*	%
Gender		
F	1658	46.3%
M	1922	53.7%
Age		
< 1 year	325	9.1%
1–3 years	723	20.2%
3–6 years	924	25.8%
6–9 years	730	20.4%
9–12 years	547	15.3%
12–16 years	331	9.2%
Children immunized against seasonal influenza virus	1217	34.0%
GAS infection(s)		
Children reporting GAS infection	725	20.3%
Children reporting > 1 GAS infection	178	24.6%

*Abbreviations: F* Female, *M* Male, *GAS* Group A streptococci infection, *n* Number

**Fig. 1:**
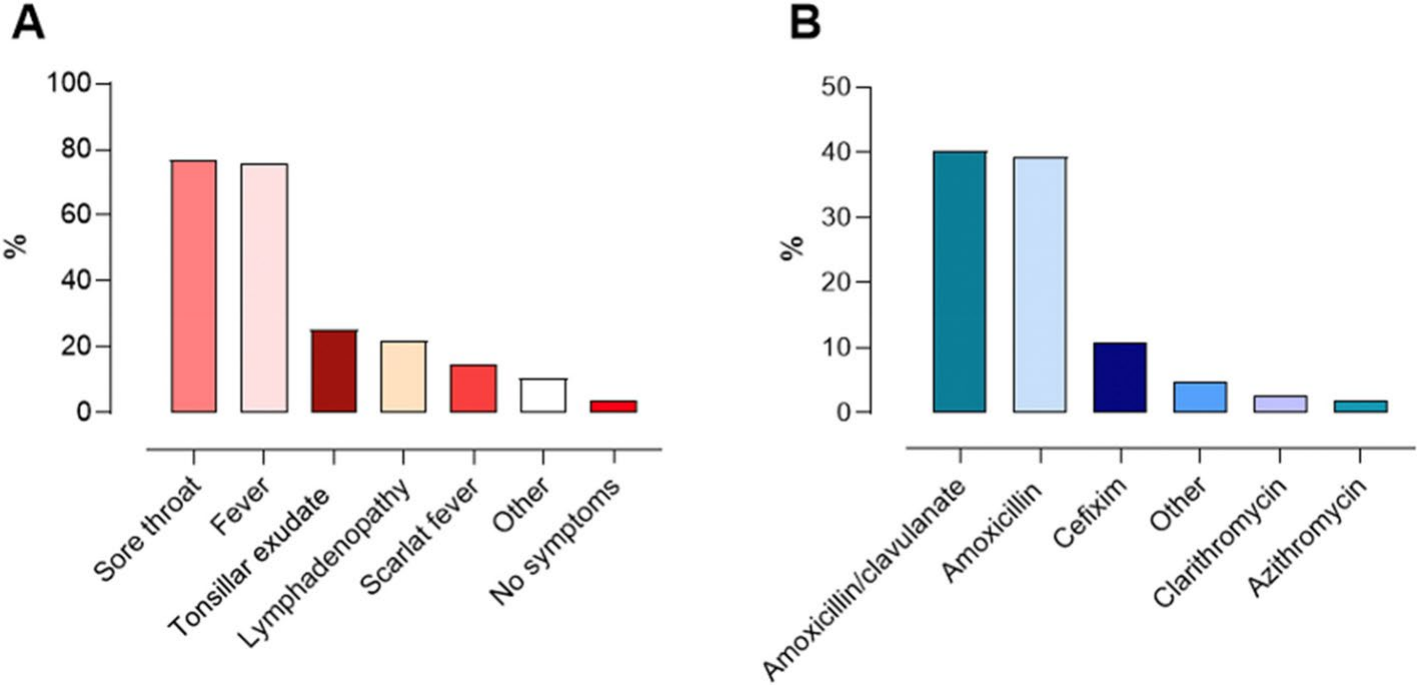
Symptoms (**A**) and treatment (**B**) of GAS infections reported

Among those with GAS infection, the main symptoms reported were sore throat (76.9%) and fever (75.2%), followed by tonsillar exudate (25.2%), lymphadenopathy (21.8%), and scarlet fever (14.7%). A single patient was hospitalized due to GAS meningitis. Approximately 4% of patients were diagnosed in the absence of symptoms (Fig. 1A). The symptoms reported according to the age of the participants are reported in Supplementary, Appendix Table A3.

Compared with children diagnosed with throat swabs, children clinically diagnosed more frequently presented with scarlet fever (29.3% vs. 15.2%, *p* = 0,003) and tonsillar exudate (34.7% vs. 24.1%, *p* = 0,03).

Eighty-nine percent of the children were treated with antibiotics (Fig. 1B). Treatment included amoxicillin/clavulanate in 40.4% of children, amoxicillin in 39.4%, and cefixime in 10.8%. Macrolides were used in 4.7%. Among those not treated, only one out of five were asymptomatic. Symptoms in those not treated with antibiotics mainly included sore throat (63%) and fever (58.1%), followed by lymphadenopathies (17.6%) and tonsillar exudate (14.9%).

Twenty-four percent of the children were reported to have more than one GAS infection (Table 2). Multiple infections were more frequently observed in children aged 3–6 years (44.4%) and 6–9 years (34.8%), whereas they were less common in children aged < 3 years and > 9 years (age < 1 year: 1.1%; 1–3 years: 10.1%; 9–12 years: 6.2%; 12–16 years: 3.4%). In this group, the frequencies of symptoms reported in the first and subsequent infections were similar, except for fever and scarlet fever which were less frequent during relapses (84% vs. 75.2%, *p* = 0.044 and 20.1% vs. 7.3%, *p* = 0.001, respectively).

**Table 2:** Symptoms reported at first and subsequent episodes in participants with more than one GAS infection

	First episode		Relapse		*P* value
	*n*	%	*n*	%	
Fever	142	84%	124	75,2%	0.044
Sore throat	137	81,1%	137	83%	0.640
Tonsillar exudate	55	32,5%	42	25,5%	0.151
Lymphadenopathy	55	32,5%	45	27,3%	0.291
Scarlet fever	34	20,1%	12	7,3%	0.001
No symptoms	6	3,6%	5	3%	0.792

When we compared children with single versus recurrent GAS infection, those with multiple episodes were more likely to report at their first episode fever (84.0% vs. 74.8%, *p* = 0.013), tonsillar exudate (32.5% vs. 23.1%, *p* = 0.014), and lymphadenopathy (32.5% vs. 16.3%, *p* < 0.0001) (Supplementary, Appendix Table A4). Notably, no difference in treatment was observed among those with a single or recurrent GAS infection.

Since bacterial and viral coinfections can affect the infection severity and because influenza itself can be a risk factor for a subsequent GAS infection, we aimed to assess the effect of immunization against seasonal influenza virus on GAS disease. Thirty-four percent of participants reported being immunized for the influenza A and B viruses, with an equal distribution of intramuscular and intranasal routes of administration. The group of children with greater vaccination coverage was aged 3–6 years. The rate of GAS infection was similar between immunized and nonimmunized children (16.9% vs. 17.6%, *p* = 0.834). Moreover, no difference in the type GAS symptoms or the occurrence of complications was observed between immunized and nonimmunized children.

## Discussion

During the recent COVID-19 pandemic, the number of circulating pathogens drastically decreased due to the lockdown and the use of masks to prevent the spread of SARS-CoV-2. Since December 2022, a strong resurgence of infections among children was observed, especially caused by respiratory pathogens such as RSV and influenza [17–19]. In this context, an increase in the numbers of GAS infections has also been reported worldwide [5, 11–15, 20].

Despite the availability of international guidelines on the diagnosis and management of GAS infection and its complications [21], the massive outbreak and recurrence of GAS have made care challenging for pediatricians.

This study aimed to investigate this phenomenon and to provide help in handling these cases, by describing the characteristics and management of nGAS infections among children living in a specific area of Italy, the Lazio region. To the best of our knowledge, this is the first Italian study to describe nGAS infections that occurred after the COVID-19 pandemic in a community outpatient setting and, importantly, the first one promoted by family pediatricians.

In children aged 0–16 years, we reported that the prevalence of GAS infection was 20.3%. The highest prevalence was recorded mainly between 3 to 6 years, a younger age than previously reported [22].

Differently from invasive infections, notification is not required for nGAS infections, making comparisons with the prevalence in Italy or in other states in the pre-COVID-19 period impossible.

Moreover, while an increase in iGAS infections has been documented in numerous studies [5, 11–15, 20], only limited data on nGAS infections are available.

In the Netherlands, in 2022, the number of general practice consultations for GAS pharyngitis in children aged 5–14 years was estimated to be approximately twice than in 2019 [23].

Similarly, an Australian study reported that the number of GAS isolates from throat swabs in the emergency department setting markedly increased in December 2022, reaching triple the mean pre-pandemic December rate. At the same time, a reduced age of onset was observed [24].

In Denmark, the number of culture-positive test results increased beginning in November 2022, peaking in January 2023 and reaching 3.5 times the incidence observed in the years 2018-19, with the greatest increase observed among children younger than 5 years [11].

In our study, the symptoms reported were nuanced, characterized mainly by fever and sore throat at the first episode and mostly by sore throat in cases of recurrence. Moreover, in most of the patients (81%), the diagnosis was confirmed by a rapid antigenic test, a test commonly used to support diagnosis because its specificity is sufficiently high to prevent unnecessary use of antibiotics [25].

It is important to emphasize that two clinical signs previously considered to characterize nGAS infection, tonsillar exudate and lymphadenopathy, have been reported only in 25.2% and 21.8% of cases, respectively, while the small group of children who did not undergo a swab test for the diagnosis, presented mainly scarlet fever and tonsillar exudate. The attenuated clinical picture can easily be confused with a viral infection, and for this reason, it is even more important to carry out microbiological tests for the correct diagnosis.

Numerous scoring systems have been developed for years considering 4 signs/symptoms (tonsillar exudate, satellite lymphadenopathy, absence of cough, and fever) [26], and age (3–14 years as more at risk) [27]. Many guide lines from different countries exists but they are characterized by several discrepancies in the diagnostic and treatment approach [21]. Among them, Italian guidelines state that none of the available scoring systems are sufficiently accurate alone to identify GAS pharyngitis with reasonable certainty [28]. Antigenic test is then important for confirming the diagnosis of symptomatic cases with high clinical scores. In contrast, a low score can be considered valid for ruling out GAS pharyngitis, in settings with no GAS epidemics and/or with a low incidence of rheumatic disease, and not proceeding with tests or treatments.

Considering the attenuated symptoms mainly reported in our study, the younger age of the subjects affected and the worldwide outbreak of GAS infection recently observed, clinical and diagnostic test criteria should be re-evaluated and discussed to avoid undiagnosed infections and subsequent related complications [22].

At the same time, using the antigenic test also in cases of mild symptoms such as feverishness, sore throat without tonsillar exudate, rhinitis and cough, increases the risk of not differentiating affected children from GAS carriers with mild symptoms due to a possible viral infection. In this scenario, the availability of a swab test to check for viral respiratory infections could be useful for differential diagnosis.

The aspect of healthy carriers should always be considered in the management of a GAS infection, especially in the suspect of a recent reinfection, as this condition could lead to misdiagnosis.

In our study, 24% of children were reported to have more than one GAS infection episode, and symptoms reported in the following infection were nuanced than symptoms reported in the first infection.

The rates of GAS carriage vary greatly depending on the population studied, ranging from 8.4 to 12.9% in high-income countries [29]. There is general agreement that carriers do not have an increased risk of complications and are unlikely to transmit GAS pharyngitis. Thus, routine antibiotic treatment and investigation of carriers are strongly not recommended [28], but should be considered in selected situations such as local outbreaks of GAS pharyngitis, iGAS disease, ARF or poststreptococcal glomerulonephritis and multiple episodes of GAS pharyngitis occurring in a family for many weeks despite appropriate treatment [30]. Moreover, carriers who develop an acute GAS infection caused by another GAS type should be treated [28]. In this framework, determining GAS type or quantifying the bacterial load could be useful, although these tests are not routinely available. In case of persistence or recurrence of symptoms compliance to therapy and evaluation of other infective causes should be performed [28].

In our survey, 89% of the children were treated with antibiotics and most of them received a β-lactamase, following the guidelines indications [28]. Indeed, penicillin or amoxicillin is the antibiotic of choice for treating GAS pharyngitis since, despite the first reported mutations that confer reduced penicillin sensitivity [2], there is no evidence of newly emerging genetic variants, and GAS remains still susceptible to β-lactamase antibiotics [5, 21].

However, since October 2022, in Europe a shortage of amoxicillin caused by manufacturing delays and production capacity issues has led to supply problems in the majority of the EU Member States. The main reason was a surge in respiratory infections that led to an increased demand for antibiotics [31]. Thus, many pediatricians prescribed amoxicillin/clavulanate but subsequently also this kind of antibiotic was difficult to find.

The use of antibiotics with a wider spectrum of activity does not confer any benefit and can lead to adverse effects [32]. An Italian study performed by family pediatricians reported that children with rapid test confirmed GAS pharyngitis, received mostly amoxicillin, while those not confirmed by swab test were equally likely to receive narrow or broad-spectrum antibiotics [33]. Similarly, in an inpatient setting, it was reported that only a minority of Italian emergency units utilize both scoring systems and rapid antigen test to manage pediatric patients with pharyngitis, considering also the low availability of the rapid antigen test in that context. Thus, almost half of the emergency units involved in that study prescribe antibiotics in children with pharyngitis without a microbiologically GAS confirmation and one over four units administer amoxicillin-clavulanate as the primary treatment for GAS pharyngitis [32]. These data highlight the importance of a rapid antigenic test availability supporting the clinical diagnosis of GAS infection to avoid inappropriate antibiotic prescriptions.

Concerning scarlet fever, in our study it was reported in 14.7% of children as a first or only manifestation and was less frequent during relapses, occurring in only 7.3% of cases. We also reported a case of meningitis due to GAS infection. Scarlet fever has an excellent prognosis when promptly treated with antibiotics such as penicillin and amoxicillin as the first-line treatment, to reduce the risk of potential complications and reduce transmission [34]. We did not observe short-term complications related to scarlet fever.

As well as for GAS pharyngitis, hypotheses for the increase in scarlet fever observed in different countries include decreased herd immunity due to COVID-19 restrictions, environmental factors such as the early start of the respiratory season and the high incidence of circulating respiratory viruses leading to possible coinfections [5, 11–15, 20].

Indeed, there have been reports of GAS coinfection with respiratory viruses, such as influenza virus and RSV, and Varicella zoster. The increase in nGAS and iGAS seems to be concurrent, particularly with the seasonal increase of influenza [13, 16]. GAS and the majority of respiratory viruses share the same seasonal pattern of infection rate, it is likely that they influence each other and have an effect on the infection severity [35]. In this context, vaccination against influenza has been shown to reduce the risk of iGAS [36].

In our study, 34% of children reported being immunized against the seasonal influenza viruses, mainly those younger than 6 years of age. This prevalence is greater than that reported in the overall Italian population (20%), possibly due to the availability of the intranasal vaccinations for kids [37]. Despite the higher rate, it is still far from the minimum coverage target of 75% for all age groups. All patients except one presented uncomplicated nGAS infections, both in the group of vaccinated and nonvaccinated for influenza, thus it was not possible to evaluate the role of flu vaccination in preventing the risk of iGAS or nGAS complications. However, considering that children, like older people, are generally more at risk of influenza and GAS complications, it is important to improve vaccination campaign and extend the vaccination coverage to protect children from coinfections that could worsen the disease, especially in frail patients, and to misdiagnose the etiology of pharyngitis.

Our study has some limitations: it is a survey in which responses were obtained by parents and were not validated by pediatricians. Moreover, the data were representative of only one region of Italy. To make the questionnaire more accessible and to obtain as many responses as possible from patients’ families, we limited the number of questions in the survey, thus we could not better define the appropriateness of diagnostic tests and treatment, especially on recurrences, nor duration of antibiotic prescriptions. However, our study also has strengths, as it the first Italian study reporting data on GAS infections in a large cohort of children evaluated by family pediatricians.

## Conclusions

To the best of our knowledge our study is the first to describe pediatric nGAS infections that occurred in an Italian region after the COVID-19 pandemic. We reported a certain prevalence of nGAS infections in children, especially those aged 3–6 years age, who were mostly characterized by low score symptoms and in most of the cases diagnosed and treated using a microbiological test as confirmatory tool. Moreover, our study showed that patients with recurrent infections experienced relapses with nuanced symptoms than the first infection.

According to these findings, the available clinical scoring systems may be not accurate enough to identify nGAS in this new clinical setting. On the other hand, new integrative microbiological tests are needed in order to identify possible viral coinfections that could confound the scenario, especially in case of recurrences. In the future it would be desirable to perform a national study to obtain more significant data and improve the clinical management of nGAS infections in children.

## Supplementary Information

Additional file 1: Table A1. Survey sent to parents or caregivers of patients aged 0–16 years including questions on Group A beta-hemolytic Streptococcus infections that occurred from January 1 to May 31, 2023. Table A2. English version of the survey. Table A3. Symptoms reported by age of participants. For those with more than one infection, only symptoms occurred at first episode were collected. Table A4. Data on children reporting more than one GAS infection at their first episode compared to data on children with a single GAS infection.

## Data Availability

Deidentified data may be made available on reasonable request to the corresponding author.

## Abbreviations

FIMP: Federazione Italiana Medici Pediatri
nGAS: non-invasive Group A beta-hemolytic streptococcus
iGAS: invasive Group A beta-hemolytic streptococcus
COVID-19: Coronavirus disease 19
RSV: respiratory syncytial virus

## References

[1] Cunningham MW. Pathogenesis of Group A Streptococcal infections. Clin Microbiol Rev. 2000;13:470–511. 10885988 10.1128/cmr.13.3.470-511.2000 PMC88944

[2] Brouwer S, Rivera-Hernandez T, Curren BF, Harbison-Price N, De Oliveira DMP, Jespersen MG, et al. Pathogenesis, epidemiology and control of Group A Streptococcus infection. Nat Rev Microbiol. 2023;21:431–47. 36894668 10.1038/s41579-023-00865-7 PMC9998027

[3] Shaikh N, Leonard E, Martin JM. Prevalence of streptococcal pharyngitis and streptococcal carriage in children: a meta-analysis. Pediatrics. 2010;126:e557–64. 20696723 10.1542/peds.2009-2648

[4] Mariani F, Gentili C, Pulcinelli V, Martino L, Valentini P, Buonsenso D. State of the art of Invasive Group A Streptococcus infection in children: a scoping review of the literature with a focus on predictors of invasive infection. Child (Basel). 2023;10(9):1472. 10.3390/children10091472 PMC10528266 37761433

[5] https://www.who.int/emergencies/disease-outbreak-news/item/2022-DON429

[6] Lee J-S, Kim S, Excler J-L, Kim JH, Mogasale V. Global economic burden per episode for multiple diseases caused by group a Streptococcus. NPJ Vaccines. 2023;8:69. 37188693 10.1038/s41541-023-00659-1 PMC10184078

[7] Beaton A, Kamalembo FB, Dale J, Kado JH, Karthikeyan G, Kazi DS et al. The American Heart Association’s call to action for reducing the global burden of Rheumatic Heart Disease: a Policy Statement from the American Heart Association. Circulation. 2020;142(20):e358–68. 10.1161/CIR.0000000000000922 33070654

[8] Nijman RG, Honeyford K, Farrugia R, Rose K, Bognar Z, Buonsenso D, et al. Presentations of children to emergency departments across Europe and the COVID-19 pandemic: a multinational observational study. PLoS Med. 2022;19:e1003974. 36026507 10.1371/journal.pmed.1003974 PMC9467376

[9] Amarsy R, Fournier S, Trystram D, Monteil C, Raynaud X, Jarlier V, et al. Decrease of hospital- and community-acquired bloodstream infections due to Streptococcus pneumoniae and Streptococcus pyogenes during the first year of the COVID-19 pandemic: a time-series analysis in Paris region. Am J Infect Control. 2023;51:475–7. 36115540 10.1016/j.ajic.2022.09.002 PMC9474397

[10] https://www.gov.uk/government/news/ukhsa-update-on-scarlet-fever-and-invasive-group-a-strep-1

[11] Johannesen TB, Munkstrup C, Edslev SM, Baig S, Nielsen S, Funk T et al. Increase in invasive group a streptococcal infections and emergence of novel, rapidly expanding sub-lineage of the virulent Streptococcus pyogenes M1 clone. Denmark. 2023;28(26):2300291. 10.2807/1560-7917.ES.2023.28.26.2300291 PMC10311951 37382884

[12] Kizil MC, Kara Y, Bozan G, Arda S, Durmaz G, Kilic O, et al. Consecutive seven serious cases with Invasive Group A Streptococcal Infections at December 2022-January 2023. Pediatr Infect Dis J. 2023;42:e254–5. 36854121 10.1097/INF.0000000000003895

[13] Lassoued Y, Assad Z, Ouldali N, Caseris M, Mariani P, Birgy A et al. Unexpected increase in Invasive Group A Streptococcal infections in Children after respiratory viruses outbreak in France: a 15-Year time-series analysis. Open Forum Infect Dis. 2023;10(5):ofad188. 10.1093/ofid/ofad188 PMC10167988 37180594

[14] Alcolea-Medina A, Snell LB, Alder C, Charalampous T, Williams TGS, Tan MKI, et al. The ongoing Streptococcus pyogenes (Group A Streptococcus) outbreak in London, United Kingdom, in December 2022: a molecular epidemiology study. Clin Microbiol Infect. 2023;29:887–90. 36925107 10.1016/j.cmi.2023.03.001 PMC10769882

[15] https://www.ecdc.europa.eu/en/news-events/increase-invasive-group-streptococcal-infections-among-children-europe-including

[16] Sultana Q, Agrawal V, Jaiswal V, Mohanty A, Sah R. Was the COVID pandemic suppressing the outbreak of scarlet fever in the United Kingdom? Correspondence. Int J Surg. 2023;109:626–8. 36906776 10.1097/JS9.0000000000000225 PMC10389450

[17] Maison N, Omony J, Rinderknecht S, Kolberg L, Meyer-Bühn M, von Mutius E, et al. Old foes following news ways?—Pandemic-related changes in the epidemiology of viral respiratory tract infections. Infection. 2024;52:209–18. 37644253 10.1007/s15010-023-02085-w PMC10811157

[18] Daniels D, Wang D, Suryadevara M, Wolf Z, Nelson CB, Suh M, et al. Epidemiology of RSV Bronchiolitis among Young Children in Central New York before and after the onset of the COVID-19 pandemic. Pediatr Infect Disease J. 2023;42:1056–62. 37725814 10.1097/INF.0000000000004101

[19] Lee SS, Viboud C, Petersen E. Understanding the rebound of influenza in the post COVID-19 pandemic period holds important clues for epidemiology and control. Int J Infect Dis. 2022;122:1002–4. 35932966 10.1016/j.ijid.2022.08.002 PMC9349026

[20] de Ceano-Vivas M, Molina Gutiérrez MÁ, Mellado-Sola I, García Sánchez P, Grandioso D, Calvo C et al. Streptococcus pyogenes infections in Spanish children before and after the COVID pandemic. Coming back to the previous incidence. Enfermedades infecciosas y microbiologia clinica (English ed). 2024;42(2):88–92. 10.1016/j.eimce.2023.04.021 37394399

[21] Pellegrino R, Timitilli E, Verga MC, Guarino A, Iacono I, Dello, Scotese I, et al. Acute pharyngitis in children and adults: descriptive comparison of current recommendations from national and international guidelines and future perspectives. Eur J Pediatr. 2023;182:5259–73. 37819417 10.1007/s00431-023-05211-w PMC10746578

[22] Miller KM, Carapetis JR, Van Beneden CA, Cadarette D, Daw JN, Moore HC, et al. The global burden of sore throat and group a Streptococcus pharyngitis: a systematic review and meta-analysis. EClinicalMedicine. 2022;48:101458. 35706486 10.1016/j.eclinm.2022.101458 PMC9124702

[23] de Gier B, Marchal N, de Beer-Schuurman I, te Wierik M, Hooiveld M, de Melker HE et al. Increase in invasive group a streptococcal (Streptococcus pyogenes) infections (iGAS) in young children in the Netherlands, 2022. Eurosurveillance. 2023;28(1):2200941. 10.2807/1560-7917.ES.2023.28.1.2200941 PMC9817208 36695447

[24] MacPhail A, Lee WJI, Kotsanas D, Korman TM, Graham M. A rise in invasive and non-invasive group a streptococcal disease case numbers in Melbourne in late 2022. Med J Aust. 2023;218:378–9. 36950836 10.5694/mja2.51909

[25] Cohen JF, Bertille N, Cohen R, Chalumeau M. Rapid Antigen detection test for group a streptococcus in children with pharyngitis. Cochrane Database Syst Reviews. 2016;7(7):CD010502. 10.1002/14651858.CD010502.pub2 PMC6457926 27374000

[26] Centor RM, Witherspoon JM, Dalton HP, Brody CE, Link K. The diagnosis of Strep Throat in adults in the emergency room. Med Decis Making. 1981;1:239–46. 6763125 10.1177/0272989X8100100304

[27] McIsaac WJ. Empirical validation of guidelines for the management of Pharyngitis in children and adults. JAMA. 2004;291:1587. 15069046 10.1001/jama.291.13.1587

[28] Chiappini E, Principi N, Mansi N, Serra A, De Masi S, Camaioni A, et al. Management of Acute Pharyngitis in Children: Summary of the Italian National Institute of Health Guidelines. Clin Ther. 2012;34:1442–e14582. 22691611 10.1016/j.clinthera.2012.04.028

[29] Oliver J, Malliya Wadu E, Pierse N, Moreland NJ, Williamson DA, Baker MG. Group A Streptococcus pharyngitis and pharyngeal carriage: a meta-analysis. PLoS Negl Trop Dis. 2018;12:e0006335. 29554121 10.1371/journal.pntd.0006335 PMC5875889

[30] DeMuri GP, Wald ER. The Group A streptococcal Carrier State Reviewed: still an Enigma. J Pediatr Infect Dis Soc. 2014;3:336–42. 10.1093/jpids/piu030 26625454

[31] https://www.ema.europa.eu/en/documents/shortage/amoxicillin-amoxicillin/clavulanic-acid-supply-shortage_en.pdf

[32] Milani GP, Rosa C, Tuzger N, Alberti I, Ghizzi C, Zampogna S, et al. Nationwide survey on the management of pediatric pharyngitis in Italian emergency units. Ital J Pediatr. 2023;49:114. 37670391 10.1186/s13052-023-01514-8 PMC10481466

[33] Barbieri E, Donà D, Cantarutti A, Lundin R, Scamarcia A, Corrao G, et al. Antibiotic prescriptions in acute otitis media and pharyngitis in Italian pediatric outpatients. Ital J Pediatr. 2019;45:103. 31420054 10.1186/s13052-019-0696-9 PMC6697973

[34] Pardo S, Perera TB. Scarlet Fever. 2024. StatPearls Publishing 29939666

[35] de Gier B, Vlaminckx BJM, Woudt SHS, van Sorge NM, van Asten L. Associations between common respiratory viruses and invasive group a streptococcal infection: a time-series analysis. Influenza Other Respir Viruses. 2019;13:453–8. 31237087 10.1111/irv.12658 PMC6692538

[36] Turner CE. Can group a streptococcus infections be influenced by viruses in the respiratory tract? Lancet Infect Dis. 2023;23:142–4. 36566769 10.1016/S1473-3099(22)00865-9

[37] http://www.salute.gov.it/portale/influenza/dettaglioContenutiInfluenza.jsp?lingua=italiano&id=679&area=influenza&menu=vuoto.

