# Supplementary material for: Group A Streptococcus infections in children and adolescents in the post-COVID-19 era: a regional Italian survey
Source: Ital J Pediatr. 2024 Sep 16;50:177. doi: 10.1186/s13052-024-01750-6 (PMC11407006; doi:10.1186/s13052-024-01750-6)
Supplement: Supplementary file 1 — Additional file 1: Table A1. Survey sent to parents or caregivers of patients aged 0–16 years including questions on Group A beta-hemolytic Streptococcus infections that occurred from January 1 to May 31, 2023. Table A2. English version of the survey. Table A3. Symptoms reported by age of participants. For those with more than one infection, only symptoms occurred at first episode were collected. Table A4. Data on children reporting more than one GAS infection at their first episode compared to data on children with a single GAS infection. [file 13052_2024_1750_MOESM1_ESM.docx]

**Additional file 1**

**Supplementary, Appendix:**

**Table A1.** Survey sent to parents or caregivers of patients aged 0-16 years including questions on Group A beta-hemolytic streptococci (GAS) infections that occurred from 1st January to 1st May 2023.

| **1) Tuo figlio ha avuto una o più infezioni da streptococco da gennaio 2023?** |
| --- |
| No nessuna |
| 1 |
| 2 |
| 3 |
| Più di 3 |
|  |
| **2) Quali sintomi ha presentato?** |
| Febbre |
| Mal di gola |
| Placche |
| Linfonodi del collo aumentati di volume |
| Scarlattina |
| Asintomatico |
| Altro |
|  |
| **3) Quali sintomi ha presentato la prima volta?** |
| Febbre |
| Mal di gola |
| Placche |
| Linfonodi del collo aumentati di volume |
| Scarlattina |
| Asintomatico |
| Altro (specificare) |
|  |
| **3) Quali sintomi ha presentato le volte successive?** |
| Febbre |
| Mal di gola |
| Placche |
| Linfonodi del collo aumentati di volume |
| Scarlattina |
| Asintomatico |
| Altro (specificare) |
|  |
| **5) Il bambino è stato ricoverato per l’infezione da streptococco?** |
| Si |
| No |
|  |
| **6) Ha eseguito un tampone faringeo?** |
| Si, test rapido |
| Si, test colturale |
| No |
|  |
| **7) Ha eseguito antibioticoterapia?** |
| Si |
| No |
|  |
| **8) Se si, specificare quale:** |
| Amoxicillina |
| Amoxicillina/acido clavulanico |
| Cefixima |
| Claritromicina |
| Azitromicina |
| Altro |
|  |
| **9) Il bambino ha eseguito la vaccinazione antinfluenzale in questa stagione?** |
| Si, Spray nasale |
| Si, Vaccino Intramuscolare |
| No |

**Table A2.** English version of the survey (for editorial purpose).

| **1) Has your child had one or more streptococcal infections since January 2023?** |
| --- |
| No |
| 1 |
| 2 |
| 3 |
| More than 3 |
|  |
| **2) What symptoms did your child present with?** |
| Fever |
| Sore throat |
| Plaques |
| Increased lymph nodes in the neck |
| Scarlet fever |
| Asymptomatic |
| Other |
|  |
| **3) What symptoms did your child first present with?** |
| Fever |
| Sore throat |
| Plaques |
| Increased lymph nodes in the neck |
| Scarlet fever |
| Asymptomatic |
| Other (specify) |
|  |
| **3) What symptoms did your child present with the following times?** |
| Fever |
| Sore throat |
| Plaques |
| Increased lymph nodes in the neck |
| Scarlet fever |
| Asymptomatic |
| Other (specify) |
|  |
| **5) Was the child hospitalized for streptococcal infection?** |
| Yes |
| No |
|  |
| **6) Has your child performed a pharyngeal swab?** |
| Yes, rapid test |
| Yes, culture test |
| No |
|  |
| **7) Has your child received antibiotic therapy?** |
| Yes |
| No |
|  |
| **8) If yes, please specify which one:** |
| Amoxicillin |
| Amoxicillin/ clavulanic acid |
| Cefixime |
| Clarithromycin |
| Azithromycin |
| Other |
|  |
| **9) Did the child get a flu vaccination this season?** |
| Yes, nasal spray |
| Yes, intramuscular vaccine |
| No |

**Table A3.** Symptoms reported by age of participants. For those with more than one infection, only symptoms occurred at first episode were collected.

|  | **n** | **Sore Throat** | | **Fever** | | **Scarlet Fever** | | **Tonsillar exudate** | | **Lymphadenopathy** | | **Meningitis** | | **Asymptomatic** | |
| --- | --- | --- | --- | --- | --- | --- | --- | --- | --- | --- | --- | --- | --- | --- | --- |
|  |  | n | % | n | % | n | % | n | % | n | % | n | % | n | % |
| **< 1 year** | 6 | 4 | 66,7% | 4 | 66,7% | 0 | 0,0% | 1 | 16,7% | 2 | 33,3% | 0 | 0,0% | 0 | 0,0% |
| **1-3 years** | 118 | 69 | 58,5% | 84 | 71,2% | 28 | 24,6% | 13 | 11,0% | 19 | 16,1% | 0 | 0,0% | 9 | 7,6% |
| **3-6 years** | 307 | 210 | 68,4% | 239 | 77,9% | 69 | 23,2% | 64 | 20,8% | 58 | 18,9% | 1 | 0,3% | 12 | 3,9% |
| **6-9 years** | 192 | 163 | 84,9% | 137 | 71,4% | 18 | 9,5% | 55 | 28,6% | 41 | 21,4% | 0 | 0,0% | 7 | 3,6% |
| **9-12 years** | 83 | 73 | 88,0% | 62 | 74,7% | 2 | 0,025 | 37 | 44,6% | 16 | 19,3% | 0 | 0,0% | 1 | 1,2% |
| **12-16 years** | 19 | 15 | 78,9% | 14 | 73,7% | 0 | 0,0% | 8 | 42,1% | 6 | 31,6% | 0 | 0,0% | 0 | 0,0% |

**Table A4.** Data on children reporting more than one GAS infection at their first episode compared to data on children with a single GAS infection.

|  | **Single GAS infection**  **n=547** | | **More than one GAS infection**  **n=178** | |
| --- | --- | --- | --- | --- |
|  | N | % | N | % |
| Sex |  |  |  |  |
| F | 243 | 44,4% | 72 | 40,4% |
| M | 304 | 55,6% | 106 | 59,6% |
|  |  |  |  |  |
| Age |  |  |  |  |
| < 1 year | 4 | 0,7% | 2 | 1,1% |
| 1-3 years | 100 | 18,3% | 18 | 10,1% |
| 3-6 years | 228 | 41,7% | 79 | 44,4% |
| 6-9 years | 130 | 23,8% | 62 | 34,8% |
| 9-12 years | 72 | 13,2% | 11 | 6,2% |
| 12-16 years | 13 | 2,4% | 6 | 3,4% |
|  |  |  |  |  |
| Antibiotic treatment | 464 | 87,5% | 153 | 93,9% |
|  |  |  |  |  |
| Symptoms |  |  |  |  |
| Sore throat | 397 | 74,6% | 137 | 81,1% |
| Fever | 398 | 74,8% | 142 | 84,0% |
| Tonsillar exudate | 123 | 23,1% | 55 | 32,5% |
| Lymphadenopathy | 87 | 16,4% | 55 | 32,5% |
| Scarlet fever | 83 | 15,6% | 34 | 20,1% |
| Gastrointestinal symptoms | 9 | 1,7% | 2 | 1,2% |
| Asthenia | 7 | 1,3% | 2 | 1,2% |
| Cephalea | 4 | 0,8% | 4 | 2,4% |
| Rhinitis | 7 | 1,3% | 1 | 0,6% |
| Cought | 6 | 1,1% | 2 | 1,2% |
| Meningitis | 1 | 0,18% | 0 | 0% |
| No symptoms | 23 | 4,3% | 6 | 3,6% |
|  |  |  |  |  |
| Diagnosis based on swab test | 466 | 87,8% | 150 | 92,0% |
| Antigenic swab test | 435 | 81,9% | 127 | 77,9% |
| Cultural swab test | 31 | 5,8% | 23 | 14,1% |
|  |  |  |  |  |
| Seasonal influenza immunization | 207 | 41,2% | 66 | 42,6% |
| Intramuscolar vaccine | 90 | 17,9% | 19 | 12,3% |
| Intranasal spray vaccine | 117 | 23,3% | 47 | 30,3% |
